# Interaction between Thalamus and Hippocampus in Termination of Amygdala-Kindled Seizures in Mice

**DOI:** 10.1155/2016/9580724

**Published:** 2016-10-17

**Authors:** Zhen Zhang, Jia-Jia Li, Qin-Chi Lu, Hai-Qing Gong, Pei-Ji Liang, Pu-Ming Zhang

**Affiliations:** ^1^School of Biomedical Engineering, Shanghai Jiao Tong University, Shanghai 200240, China; ^2^Department of Neurology, Ren Ji Hospital, School of Medicine, Shanghai Jiao Tong University, Shanghai 200127, China

## Abstract

The thalamus and hippocampus have been found both involved in the initiation, propagation, and termination of temporal lobe epilepsy. However, the interaction of these regions during seizures is not clear. The present study is to explore whether some regular patterns exist in their interaction during the termination of seizures. Multichannel in vivo recording techniques were used to record the neural activities from the cornu ammonis 1 (CA1) of hippocampus and mediodorsal thalamus (MDT) in mice. The mice were kindled by electrically stimulating basolateral amygdala neurons, and Racine's rank standard was employed to classify the stage of behavioral responses (stage 1~5). The coupling index and directionality index were used to investigate the synchronization and information flow direction between CA1 and MDT. Two main results were found in this study. (1) High levels of synchronization between the thalamus and hippocampus were observed before the termination of seizures at stage 4~5 but after the termination of seizures at stage 1~2. (2) In the end of seizures at stage 4~5, the information tended to flow from MDT to CA1. Those results indicate that the synchronization and information flow direction between the thalamus and the hippocampus may participate in the termination of seizures.

## 1. Introduction

Epilepsy is a kind of chronic neurological disorder characterized by highly synchronized abnormal discharge of neurons. Epileptic seizures can cause serious physiological and psychological damage to patients. Due to that the seizures involve complex interactions across some regions of the brain; insights into the interrelations across some key areas during the evolution of seizures may help us to understand the pathogenesis of epilepsy as well as improve the therapy of epilepsy [1].

Temporal lobe epilepsy (TLE) is the most common type of focal epilepsy in clinic [2]. Recent studies showed that the TLE involves some encephalic regions such as the hippocampus and some of its neighboring regions including the thalamus, amygdala, and entorhinal cortex [3, 4]. Cendes et al. suggested that the most frequent neuropathological change in TLE patients was the hippocampal sclerosis [5]. Many studies have reported that the abnormal electrical activities were often detected in the hippocampus of TLE patients [6–8]. Moreover, during recent years, some researches have reported that the thalamus also plays an important role in TLE. Some studies have suggested that the thalamus is a key part in the initiation and propagation of TLE seizures [9–11]. Bertram found that the cornu ammonis 1 (CA1) of hippocampus and mediodorsal thalamus (MDT) were both involved in amygdala-kindled seizures [1, 3]. Andrade et al. showed that electrically stimulating MDT could remedy TLE [12]. Bertram et al. suggested that the seizure duration would be significantly decreased if strengthening the activity of GABAergic neurons in the MDT in the hippocampus-kindled mice [13]. All of those results are calling for more investigations on the interaction between the thalamus and the hippocampus in the termination of seizures.

To investigate the time-varying interactions between different brain areas, a number of methods, such as coupling index (CI) [14, 15] and directionality index (DI) [16, 17], have been suggested. The CI and DI represent the level of synchronization and information flow direction between different brain areas. Due to the nonlinear property of epileptic discharges, the nonlinear methods are preferred in analyzing the interactions between different brain areas during seizures [18]. The information-based method is a representative of nonlinear algorithms, which has been widely applied to analyze the epileptic discharges [10, 18, 19]. Mutual information (MI) is a useful method to study the synchronization between two time series [14]. Stam has demonstrated that MI is more robust for estimation of the changes in neural electrical activity than a linear method of spectral power analysis [20]. Frasch et al. have suggested that MI is suited to measure changes in synchronization of the neural electrical activities in different brain areas, because it is not an amplitude dependent measure [21]. Conditional mutual information (CMI) is an information theory based method to determine the information flow direction between two time series [22]. Recently, the permutation information approach and CMI were integrated, which was called permutation conditional mutual information (PCMI) [23], to estimate the coupling direction between different neuron populations [11]. The stimulation results have shown that this method is superior to CMI for measuring the characteristics of coupling direction between neuron populations [24]. Mi et al. have demonstrated that PCMI can effectively estimate the directionality of local field potentials (LFPs) between CA1 and CA3 in rats [25]. By utilizing MI and PCMI to analyze the LFPs recorded from the hippocampus and the thalamus, the interactions between them in the termination of seizures can be investigated.

The present paper is organized as follows.  Section 2 presents the scheme of experimental data acquisition and briefly introduces the algorithms for estimating the MI and PCMI. The results of how the MI and PCMI vary during the evolution of seizures are presented in Section 3. The conclusion is provided in Section 4.

## 2. Materials and Methods

### 2.1. Data Acquisition

#### 2.1.1. Animals

Adult (3 to 5 months) male C57BL/6 mice were used in our experiments. The mice were housed in individual cages with food and water ad libitum and kept in a 12 h light/dark cycle. All animal experiments were approved by the Ethic Committee, School of Biomedical Engineering, Shanghai Jiao Tong University. All efforts were made to minimize the number of animals used and their suffering.

#### 2.1.2. Electrophysiological Recordings

Experimental procedures were described in our previous report [26]. In brief, two recording tetrodes were implanted in the MDT of thalamus (with bregma as the reference, anteroposterior (AP), −1.2 mm; mediolateral (ML), −0.6 mm; dorsoventral (DV), −3.1 mm) and the CA1 of right hippocampus (AP, −1.2 mm; ML, −0.6 mm; DV, −1.7 mm), and one stimulation bipolar electrode was implanted in the right basolateral amygdala (BLA, AP, −1.2 mm; ML, −2.6 mm; DV, −4.9 mm). The recording tetrode was formed of four twisted polyester insulated nickel-chrome alloy wires (diameter, 13 *μ*m; California Fine Wire Co., USA). The stimulation electrode was composed of two stainless steel channels (diameter, 50 *μ*m; AM System Co., USA). The recording tetrodes were gilded before being implanted to the target regions to make the impedance in the range 0.5~1.0 MΩ.

Seven days after the electrodes' implantation, a multichannel in vivo recording system (Plexon Co., USA) and an electrical stimulator (NIHON KOHDEN Co., Japan) were connected to the electrodes. For each mouse, the amplitude of the pulses for stimulating BLA was set as 60 *μ*A (1 s train containing 60 Hz unidirectional pulses) at the first time and increased by 20 *μ*A every 10 min until the duration of the afterdischarge (AD) recorded in the BLA was longer than 5 s; then this stimulating amplitude was used in the further kindling process in this mouse. After the amplitude of the current pulses for stimulating was assigned, the kindling acquisition was achieved by stimulating BLA twice daily with subconvulsive electrical stimulations at a 4-hour interval. During the kindling process, the LFPs in the MDT and CA1 were recorded at a sampling frequency of 1000 Hz and stored for offline analysis. Racine's rank standard was employed to classify the stage of behavioral responses (stage 1~5) [27]. The mouse in which three consecutive stage 4~5 seizures were induced was regarded as a fully kindled mouse [28]. After the electrophysiological recording finished, the histology check was carried out to inspect whether the electrodes were implanted in the correct positions. If the mouse was fully kindled and passed the histology check, three seizures at stage 1~2 (the first one, the middle one, and the last one) and two seizures at stage 4~5 (the last two) of this mouse were selected for further analysis. Seven fully kindled mice were recruited in this study.

### 2.2. Data Analysis

#### 2.2.1. Data Preprocessing

After the LFPs in the MDT and CA1 were collected, notch filtering was performed to filter out the 50 Hz power noise. Due to that the distance between four wires in the recording tetrodes was about 15~25 *μ*m; LFP signals recorded by the wires of a tetrode were highly similar. Therefore, the mean value of LFP signals among the four wires was used to represent the LFP signal recorded by the tetrode. In the following text, the term of LFP in the calculation of coupling index and directionality index refers to the mean value.

#### 2.2.2. Coupling Index-MI

The MI quantifies the shared information between time series based on information theory and is used as the measure of synchronization between those time series [18, 29]. The MI has the maximum value when the two time series are identical, and it is zero when one system is completely independent of the other [29].

Before calculating the MI value, the probability distribution functions (PDFs) of the time series should be calculated. In this study, the permutation information approach [23, 30] was applied to calculate the PDFs of time series. Given a time series *X* = (*x*_1_, *x*_2_, *x*_3_,…, *x*_*L*_) which has *L* data points, the PDF of *X* can be calculated by the following steps. Firstly, set a lag *τ* to sample the data to build the vectors (*τ* = 1 represents continuous sampling, *τ* = 2 represents dislodging 1 point, and so on) and set an order number *m* of vectors (i.e., the number of points in each vector); the vector composed of *m* points can be arranged into *N* = *m*! = 1*∗*2*∗* ⋯ *∗m* possible permutation patterns *π*_*I*_  (*I* = 1,2,…, *N*); then we get the vectors *V* = (*v*_1_, *v*_2_,…, *v*_*L*−(*m* − 1)*∗τ*_) from the time series *X*, where *v*_1_ = (*x*_1_, *x*_2_,…, *x*_*m*_), *v*_2_ = (*x*_2_, *x*_3_,…, *x*_*m*+1_), and so on (when *τ* = 1); thirdly, count the occurrences of each pattern *π*_*I*_  (*I* = 1,2,…, *N*) in the time series which is denoted as *N*(*π*_*I*_); finally, the probability density distribution *P*(*π*_*I*_) is inferred by the following equation:

(1) $$\begin{array}{c} P\left(\;\pi_{I}\;\right)=\frac{N\left(\pi_{I}\right)}{L-\left(m-1\right)*\tau };\;I=1,2,\ldots ,N. \end{array}$$

The LFPs recorded from CA1 and MDT are denoted as *X* and *Y*, respectively. The PDFs of *X* and *Y* are denoted as *p*(*π*_*x*·*i*_) and *p*(*π*_*y*·*j*_). The joint probability functions of *X* and *Y* are denoted as *p*(*π*_*x*·*i*_, *π*_*y*·*j*_). The conditional probability function of *X* given *Y* is denoted as *p*(*π*_*x*·*i*_∣*π*_*y*·*j*_).

Based on Shannon's entropy, the entropy of *X* and *Y* is defined as

(2) HX=−∑i=1Nlog⁡pπx·i,HY=−∑j=1Nlog⁡pπy·j.

The conditional entropy of *X* by *Y* (*H*(*X*∣*Y*)) is defined as

(3) $$\begin{array}{c} H\left(\;X\;|\;Y\;\right)=-\overset{N}{\underset{i=1}{\sum }}\;\overset{N}{\underset{j=1}{\sum }}p\left(\;\pi_{x\cdot i},\pi_{y\cdot j}\;\right)\log p\left(\;\pi_{x\cdot i}\;|\;\pi_{y\cdot j}\;\right). \end{array}$$

The joint entropy of *X* and *Y* is defined as

(4) $$\begin{array}{c} H\left(\;X,Y\;\right)=-\overset{N}{\underset{i=1}{\sum }}\;\overset{N}{\underset{j=1}{\sum }}p\left(\;\pi_{x\cdot i},\pi_{y\cdot j}\;\right)\log p\left(\;\pi_{x\cdot i},\pi_{y\cdot j}\;\right). \end{array}$$

The normalized mutual information of *X* and *Y* is defined as

(5) $$\begin{array}{c} I\left(\;X;Y\;\right)=\frac{\left(H\left(X\right)-H\left(X\;|\;Y\right)\right)}{H\left(X,Y\right)}. \end{array}$$

In the application of permutation information approach, the value of *τ* corresponding with the maximal value of mutual information between *X* and *Y* was decided as *τ*_opt_ [24]. *m* was set to be 3.

#### 2.2.3. Directionality Index-PCMI

The permutation conditional mutual information (PCMI) is used to estimate the coupling direction between two time series based on the information theory [23]. After the PDFs of *X* and *Y* were estimated by the permutation information approach mentioned in Section 2.2.2, the PCMI between *X* and *Y* was calculated by the following equations:

(6) IX→Yδ=HX ∣ Y+HYδ ∣ Y−HX,Yδ ∣ Y,IY→Xδ=HY ∣ X+HXδ ∣ X−HY,Xδ ∣ X,

where *I*_*X*→*Y*_^*δ*^(*I*_*Y*→*X*_^*δ*^) means the information quantity transferred from *X* to *Y* (or *Y* to *X*) when lagging *Y* behind *δ* steps as *Y*_*δ*_ (or lagging *X* behind *δ* steps as *X*_*δ*_).

The information that is transferred from *X* to *Y* (or *Y* to *X*) is defined as

(7) IX→Y=∑δ=δ1δ2IX→Yδδ2−δ1+1,IY→X=∑δ=δ1δ2IY→Xδδ2−δ1+1,

where *δ*_1_ and *δ*_2_ are the minimal and maximal lagging steps, respectively. *I*_*X*→*Y*_ and *I*_*Y*→*X*_ are averaged over a range of [*δ*_1_, *δ*_2_] to decrease the estimation fluctuations. *δ*_1_ cannot be less than *m* according to Bahraminasab et al.'s study [23]. In this study, *δ*_1_ and *δ*_2_ were 3 ms and 20 ms, respectively.

The directionality index is defined as the following equation:

(8) $$\begin{array}{c} D_{XY}=\frac{I_{X\to Y}-I_{Y\to X}}{I_{X\to Y}+I_{Y\to X}}. \end{array}$$

The value of *D*_*XY*_ ranges from −1 to 1. *D*_*XY*_ > 0 means that the information flows from *X* to *Y*, and vice versa. *D*_*XY*_ = 0 means that the interactions between *X* and *Y* are symmetrical.

In our study, moving windows were used to analyze the data. The number of data points in the moving window was set to 2000 sample points corresponding to 2 s and shifted forward in 0.5 s steps.

#### 2.2.4. Statistical Analysis

Statistics were performed using Matlab (version 7.0.0, The MathWorks, Inc., Natick, MA, USA). Results were presented as mean ± SEM. One-way analysis of variance (ANOVA) was used for comparison among multiple groups, with *p* < 0.05 indicating significant difference.

## 3. Result and Discussion

### 3.1. LFPs Recorded in CA1 and MDT during Amygdala-Kindled Seizures

**Figure 1:**
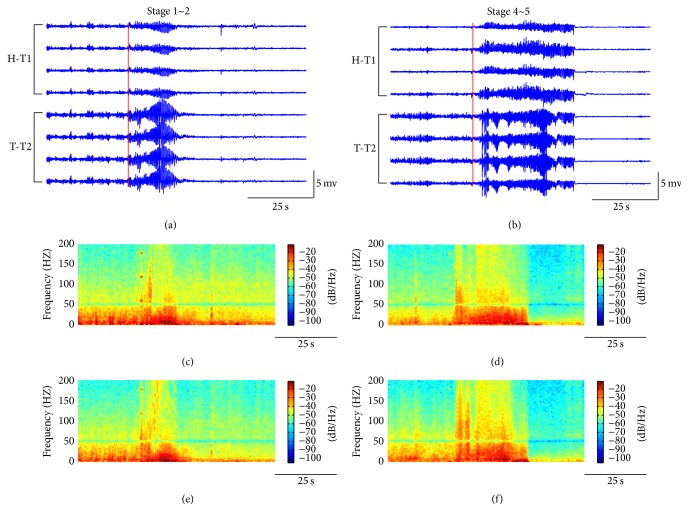
LFPs and the corresponding power spectral of seizures in one example mouse. In (a) and (b), the upper four traces represent the LFPs recorded from four channels in the tetrode T1 implanted in CA1 of hippocampus; the lower four traces represent the LFPs recorded from four channels in the tetrode T2 implanted in MDT of thalamus; the red vertical lines represent the ending time of stimulation process. In (c) and (d), power spectral of LFPs from the first channels (the top lines) in T1 in (a) and (b), respectively. In (e) and (f), power spectral of LFPs from the first channels in T2 in (a) and (b), respectively.

Neural activities were simultaneously recorded from CA1 of hippocampus and MDT of thalamus during the seizures from 7 fully kindled mice. Typically, seizures initiated immediately after electrically stimulating BLA for 1 s and manifested both behaviorally and electrographically. The epileptiform activities were observable in the hippocampus and the thalamus after the electrical stimulation (Figures 1(a) and 1(b)), and the power of LFPs was raised immediately over a wide frequency band, including theta, alpha, beta, and gamma activity (Figures 1(c)–1(f)). Because the 50 Hz power noises were filtered out, the power density spectral showed a low-power frequency band around 50 Hz.

### 3.2. Dynamics of the Synchronization between CA1 and MDT

**Figure 2:**
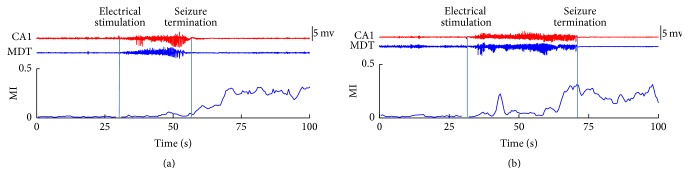
Representative results of the MI between CA1 and MDT in one seizure at stage 1~2 (a) and in one seizure at stage 4~5 (b). In (a) and (b), the upper part represents the LFPs in CA1 (red) and MDT (blue) and the lower part represents the MI between CA1 and MDT. The MI values during the stimulation time were deleted to wipe off the disturbance of the stimulus artifact. The blue vertical lines represent the ending time of stimulation process (left) and the time of the seizure termination (right).

The MI was used here as the CI to analyze the dynamic synchronization between the hippocampus and thalamus during seizures. The larger MI value means the higher level of the synchronization between CA1 and MDT. Figures 2(a) and 2(b) show the representative results of MI during one seizure at stage 1~2 and one other seizure at stage 4~5, which were captured in one mouse. The results showed that the synchronization between CA1 and MDT increased substantially after the termination of the seizure at stage 1~2, whereas it increased substantially before the termination of the seizure at stage 4~5. The similar results were also observed in the other seizures at stage 1~2 and seizures at stage 4~5 from 7 mice recruited in this study.

**Figure 3:**
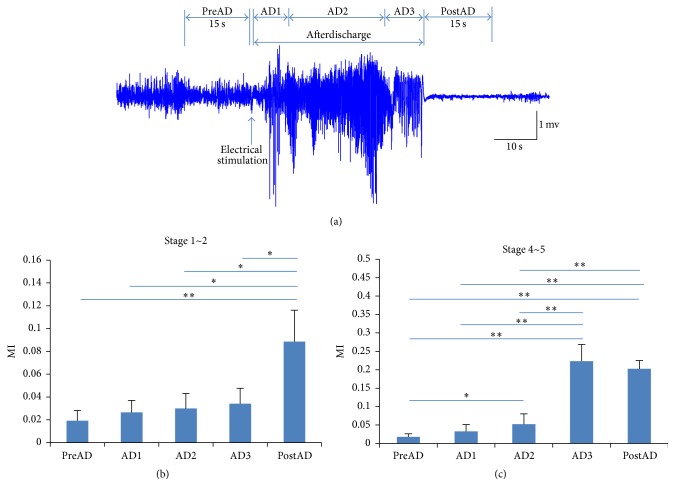
Statistics analysis results of MI. (a) Definition of time periods. The preseizure time period (PreAD) contains data in 15 s before the electrical stimulation, and the postseizure period (PostAD) contains data in 15 s after the seizure termination. The AD period was divided into three parts (AD1, AD2, and AD3). AD1 period contains the first one-fifth of the AD period. AD2 period contains the middle three-fifths of the AD period. AD3 period contains the last one-fifth of the AD period. In (b) and (c), statistical analysis results of MI in stage 1~2 group and stage 4~5 group (one-way ANOVA; stage 1~2: *n* = 21; stage 4~5: *n* = 14; ^*∗*^ *p* < 0.05, ^*∗∗*^ *p* < 0.01). Error bar means standard error.

The statistics analysis results across all of the seizures (21 seizures at stage 1~2 and 14 seizures at stage 4~5) from 7 mice are illustrated in Figure 3. As shown in Figure 3(a), five periods are defined. The preseizure time period (PreAD) contains 15 s before the electrical stimulation, and the postseizure period (PostAD) contains 15 s immediately after the seizure termination. The AD period was divided into three parts. The first part is the initiation period of seizure (AD1), containing one-fifth of the AD. The second part is the middle part of the seizure (AD2), containing three-fifths of the AD. The third part is the end of the seizure (AD3), containing one-fifth of the AD. As shown in Figure 3(b), in seizures at stage 1~2, MI did not increase significantly in AD periods (AD1, AD2, and AD3) compared with PreAD period, whereas it increased significantly in PostAD period compared with PreAD (*p* < 0.01, *n* = 21) and AD (*p* < 0.05, *n* = 21); in seizures at stage 4~5, MI of AD2 was significantly larger than that of PreAD (*p* < 0.05, *n* = 14), and MI of AD3 and PostAD were significantly larger than those of PreAD, AD1, and AD2 (*p* < 0.01, *n* = 14). There was no statistical difference between the other pairs.

Those statistical results confirmed that the synchronization between CA1 and MDT remained at a low level during the seizures at stage 1~2, whereas it increased after seizure termination. In seizures at stage 4~5, the synchronization between CA1 and MDT started to increase at the middle part of seizures, further increased in the end of seizures, and lasted for a period of time after seizure termination. The different dynamic changes of synchronization between seizures at stage 1~2 and seizures at stage 4~5 may be ascribed to different neural circuits involved [31].

Synchrony measures the relation between the temporal structures of the signals regardless of signal amplitude [15]. In this study, MI was used to detect the dynamic synchronization between the LFPs recorded from CA1 and MDT during amygdala-kindled seizures. The LFPs recorded from CA1 and MDT are considered to be synchronous if they have the shared information. Coulter et al. demonstrated that the seizure onset zone was the kindled area during the initial stage of kindling process in mice [32]. Schindler et al. reported that the epileptiform discharge propagated from the seizure onset zone to different cortical areas with different time delays [33]. Due to different time delays for epileptiform discharge propagating from the kindled BLA to CA1 and MDT, the low level of synchronization was observed during the entire seizure. After seizure termination, the synchronization between CA1 and MDT increased to a higher level and lasted for 150 s~200 s, suggesting that the coupling strength between neuron populations which were involved in seizures may continue to strengthen after the seizure termination, which was consistent with our previous study in rat hippocampal slices [34].

Some studies have shown that stimulating some areas (hippocampus, thalamus, etc.) which were anatomically connected with amygdala could also induce high stage seizures in fully kindled mice [32, 35]. Those studies suggested that when the animal model was fully kindled, the seizures may arise from multiple, distributed regions [32]. The areas involved in seizures may receive input from different seizure onset zones. Consequently, the low level of synchronization during the initiation period in seizures at stage 4~5 may be attributed to multiple, distributed seizure onsets. In the end of seizures at stage 4~5, the behavior response of kindled mice became much stronger than the initiation period, which means the seizures evolved into generalized seizures. Indic and Narayanan demonstrated that the epileptiform discharges recorded from different brain areas during generalized seizures are more correlative than that during partial seizures [36], suggesting that the level of synchronization between different brain areas during generalized seizures is higher than that during partial seizures. The enhancement of synchronization observed during the end period in seizures at stage 4~5 in this study may be attributed to the fact that the seizures transmit from partial seizures into generalized seizures.

In a traditional concept, the level of synchronization during seizures is always higher than normal or interictal period and the seizure termination may be induced by desynchronization [37, 38]. However, our results indicated that high level of synchronization between the thalamus and hippocampus was observed towards the end of seizures at stage 4~5. In recent years, several studies have reported that the hypersynchronous phenomenon was observed towards the end of seizures, which are consistent with our results. Li et al. indicated that the phase synchronization between the LFPs recorded from the hippocampus and thalamus enhanced before seizure termination in seizures at stage 3~5 in amygdala-kindling mice [26]. Guye et al. found that the synchronization between the thalamus and temporal lobe structures tended to be particularly high in the end of seizures in patients with focal epilepsy [39]. Sobayo et al. showed that a high level of phase synchronization in frequency band between 130 Hz and 160 Hz appeared between the thalamus and focal hippocampus as seizures naturally terminated in a kainic acid rat model of TLE [40]. Jiruska et al. demonstrated that the desynchronization was often observed preceding seizures or during their early stage; in contrast, high level of synchronization was observed towards the end of seizures [41]. All those studies suggest that there are complex and variable interactions between different neuron populations during the seizures.

Since the enhancement of synchronization during the final stage of the seizure was observed across different in vitro and in vivo models and human seizures [40], one question “synchronization causes seizure termination or seizure termination implies synchronization?” should be asked [42]. Sobayo et al. found that the multisite deep brain stimulation applied at the frequency which achieved the maximum phase synchronization between some sites when seizures naturally terminated can suddenly terminate seizures in a chronic rat limbic epilepsy model [43]. This study supported the fact that synchronization might be the cause of seizure termination. A series of in vivo experiments performed in cats under ketamine-xylazine anaesthesia, including multisite intracellular recordings [44] and extracellular neuronal [45] and local field potential recordings [46], showed that the increase of synchronization during seizures promoted the recruitment of neurons, and seizures terminated when all the affected neuron populations are highly synchronous. As a possible mechanism, the authors proposed that high level of depolarization of neurons during the full-blown seizures activated the hyperpolarizing potassium currents, which suppressed the depolarizing influence of ionic currents. As this effect occurs, it results in seizure termination across the entire synchronized neuron populations [44].

Several studies have demonstrated that the epileptic seizure is not a monolithic state [11, 41]. Schindler et al. found that the correlation of multichannel EEG decreased during the first half of the seizures while it increased before the seizure termination in patients with pharmacoresistant focal epilepsy [33]. In a smaller spatial scale, Warren et al. showed that the LFP synchrony between the seizure generating region and the other brain regions was lower in epilepsy patients than in control patients in the seizure initiation period [47]. In a much smaller scale, Truccolo et al. demonstrated that the neuronal spiking activity in humans was highly heterogeneous during the initiation and spreading of seizures while becoming homogeneous towards the seizure termination [48]. Those studies demonstrated that the epileptic network was functional disconnected during the initiation period of seizures but functional connected towards the seizure termination. Since the synchronization reflects a stable functional relationship [47], the evidence might give an explanation for the dynamic synchronization during seizures in our research.

### 3.3. Dynamics of the Directionality Index between CA1 and MDT

**Figure 4:**
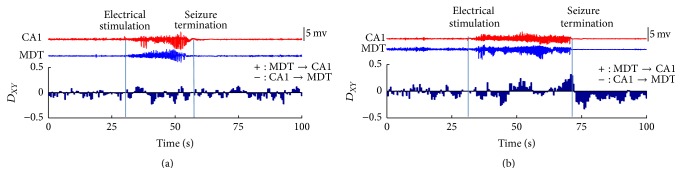
Representative results of the *D*_*XY*_ between CA1 and MDT in one seizure at stage 1~2 (a) and in one seizure at stage 4~5 (b). In (a) and (b), the upper part represents the LFPs in CA1 (red) and MDT (blue) and the lower part represents the *D*_*XY*_ between CA1 and MDT. The *D*_*XY*_ values during the stimulation time were deleted to wipe off the stimulus artifact caused by electrical stimulation. The blue vertical lines represent the ending time of stimulation process (left) and the time of the seizure termination (right).

To assess the information flow direction between two neuron populations in CA1 and MDT, the directionality index (*D*_*XY*_) based on PCMI was computed using LFPs of the two areas. A positive *D*_*XY*_ value means that the information mainly flows from MDT to CA1, and vice versa. Figures 4(a) and 4(b) show representative results of *D*_*XY*_ during one seizure at stage 1~2 and one other seizure at stage 4~5, which are the same as the two seizures shown in Figure 2. The results showed that the information mainly flowed from MDT to CA1 in the end of seizures at stage 4~5. This result was also observed in the other seizures at stage 4~5 from 7 mice recruited in this study. For the seizures at stage 1~2, we did not find consistent results.

**Figure 5:**
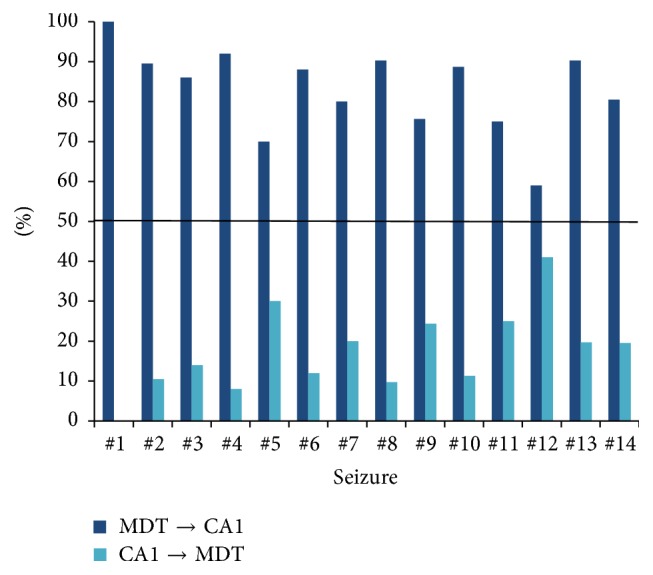
Percentages of coupling direction “MDT → CA1” and “CA1 → MDT” during the AD3 period in 14 seizures at stage 4~5. The black solid line marks the threshold of 50%.

For each period defined in Figure 3(a) and for each seizure in 21 seizures at stage 1~2 and 14 seizures at stage 4~5, we defined the percentage of *D*_*XY*_ > 0 as the percentage of information flow from MDT to CA1 and the percentage of *D*_*XY*_ < 0 as the percentage of information flow from CA1 to MDT, respectively. The percentages of coupling direction “MDT → CA1” and “CA1 → MDT” during the AD3 period in 14 seizures at stage 4~5 are illustrated in Figure 5. As shown in the figure, the information mainly flowed from MDT to CA1 in all cases. We did not find consistent results in the other periods in the seizures at stage 4~5 as well as all periods in 21 seizures at stage 1~2 (data not shown). Since the switch of information flow direction from MDT to CA1 was only consistently observed prior to seizure termination at stage 4~5 but not for the other periods, this switch might be related to seizure termination.

Bertram has shown that there are fibre connections between MDT and CA1, and those two areas are both involved in the neural circuit of TLE [3]. Previous studies have shown that the MDT is a key node in limbic seizure circuits [9]. Bertram et al. suggested that the seizure duration in CA1 would be significantly decreased if strengthening the activity of GABAergic neurons in the MDT in the hippocampus-kindled mice [13]. Zhang and Bertram showed that electrically stimulating the MDT can largely suppress the seizure duration in CA1 in the hippocampus-kindled rats of limbic epilepsy [49]. Those studies regulated the MDT by pharmacological or electrical methods and effectively reduced the seizure duration in CA1, suggesting that MDT might be an important region for seizure termination. In this study, we found that the MDT became the information source to CA1 during the end period of seizures at stage 4~5. This phenomenon may be caused by the fact that the epileptiform activity propagated from MDT to CA1 before the seizure termination. The switch of information flow direction in the end period of seizures at stage 4~5 may be an emergent self-regulatory mechanism for the seizure termination.

When comparing the results of MI and *D*_*XY*_, we found that in AD3 periods of seizures at stage 4~5 the synchronization between MDT and CA1 increased substantially (Figure 3(c)), and the information flowed from MDT to CA1 (Figure 5). The results suggested that there might be relevance between synchronization and information flow direction in MDT and CA1 in the end of seizures at stage 4~5. Both the enhancement of synchronization and the information flow direction from the MDT to CA1 may be related to seizure termination.

Studies have shown that the multiple nucleus of the thalamus is involved in the neural circuits of epilepsy [3]. Nail-Boucherie et al. showed that enhancing the glutamate activity of parafascicular nucleus could significantly suppress the paroxysmal discharges in rats with generalized absence seizures [50]. Nanobashvili et al. found that electrical stimulation of the reticular nucleus suppressed the limbic motor seizures in hippocampus-kindled rats [51]. Recent studies found that electrical stimulation of the anterior nucleus of the thalamus (ANT) could be an advisable method for seizure control in clinic [52]. Hamani et al. indicated that electrical stimulation of the ANT could reduce the seizure frequency in status epilepticus rat models [53]. Liu et al. found that the electrical stimulation of ANT caused decrease in concentrations of glutamate and increase in GABA in hippocampus in epileptic rat models [54]. Wang et al. showed that low-frequency stimulation in ANT could decrease the frequency of high-frequency oscillations and interictal spikes in hippocampus in kainate mouse model [55]. Those studies have modulated different nucleus of the thalamus and suppressed the seizure with high efficiency, suggesting that the thalamus is a key node in the neural circuits of absence epilepsy and TLE.

In recent years, the role of the thalamus in seizure termination has attracted more and more attention [1, 12]. Evangelista et al. indicated that thalamus drives mesial temporal lobe before the seizure termination in human mesial temporal lobe seizures [56]. In our study, the information mainly flowed from MDT to CA1 in the end of amygdala-kindled seizures at stage 4~5. Our result indicates that the thalamus drives the hippocampus during the end period of seizures at stage 4~5 to some extent. Bertram has indicated that the role of thalamus is to drive the target areas into excitation during limbic seizures, which promoted the synchronization between involved neuron populations [1]. In this study, the drive effect from the thalamus to hippocampus may promote the synchronization which caused seizure termination, which indicated that the thalamus may play a role in the termination of amygdala-kindled seizures. However, in this study, the electrode only recorded neural activities in MDT in the experiment. We speculated that the MDT may play a role in the neural circuits of TLE based on our computational results, but we cannot determine whether the MDT plays the most direct role in the neural circuits. It still needs to be investigated in our future work.

## 4. Conclusion

In this paper, computational methods were used to analyze LFPs to reveal the characteristics which might not be accessed by the examination of the waveforms of neural signals. Although we cannot draw a strong conclusion only by the mathematical results, we provide a new perspective to understand the evolution of amygdala-kindled seizures in mice. We found that the synchronization between the CA1 and MDT increased to a higher level after the termination of seizures at stage 1~2, whereas it increased before the termination of seizures at stage 4~5. Moreover, we found that the information mainly flowed from MDT to CA1 in the end of seizures at stage 4~5. Our results indicate that the synchronization and information flow direction between the thalamus and the hippocampus may participate in the termination of seizures. From a therapeutic perspective, researches on the interaction between thalamus and hippocampus in the termination of seizures may open new therapeutic methods for promoting seizure termination and provide a deeper understanding of the nature of seizure evaluation. However, we still have many jobs to further confirm and improve our results.
